# The Extrinsic Coagulation Pathway: a Biomarker for Suicidal Behavior in Major Depressive Disorder

**DOI:** 10.1038/srep32882

**Published:** 2016-09-08

**Authors:** Yongtao Yang, Jin Chen, Chengyu Liu, Liang Fang, Zhao Liu, Jing Guo, Ke Cheng, Chanjuan Zhou, Yuan Zhan, Narayan D. Melgiri, Liang Zhang, Jiaju Zhong, Jianjun Chen, Chenglong Rao, Peng Xie

**Affiliations:** 1 Department of Neurology, the First Affiliated Hospital of Chongqing Medical University, Chongqing, China; 2 Chongqing Key Laboratory of Neurobiology, Chongqing, China; 3 Institute of Neuroscience and the Collaborative Innovation Center for Brain Science, Chongqing Medical University, Chongqing, China; 4 Department of Neurology, Yongchuan Hospital of Chongqing Medical University, Chongqing, China

## Abstract

Although an association between major depressive disorder (MDD) and suicide exists, most depressed patients never attempt suicide. An improved understanding of the factors contributing to suicidal risk in MDD can provide direction for suicide predictor development. In MDD suicide attempters (MDD-SA), MDD non-attempters (MDD-NA), and healthy controls (HC) (n = 12 each group), complementary plasma proteomics identified 45 differential proteins mapped to coagulation and inflammation, 25 of which underwent Western blotting. In another cohort including antidepressant-treated patients (n = 49 each group), seven additional extrinsic pathway proteins were selected for ELISA. Two inflammatory proteins and eight coagulatory proteins demonstrated alterations in MDD-SA relative to MDD-NA and HC. Applying a relative mass-action ratio, MDD-SA subjects displayed a higher relative prothrombinase activity than MDD-NA subjects, while healthy controls displayed higher relative prothrombinase activity than both MDD-SA and MDD-NA subjects. Consistent with our human findings, we found that heparin treatment significantly increased forced swimming test (FST) immobility time in rodents. MDD, independent of suicidality, is associated with a proinflammatory state accompanied by a hypothrombotic state. Suicidal behavior in MDD is associated with a more pronounced proinflammatory and prothrombotic phenotype accompanied by extrinsic pathway activation, revealing an extrinsic pathway biomarker that can be applied in predicting and monitoring suicidal risk.

Multiple studies have established a strong association between major depressive disorder (MDD, major depression) and suicidal behavior^1^ with the lifetime mortality risk from suicide in the MDD population estimated at 3.4%^2^. Although approximately 60% of all suicides occur in depressed individuals, most depressed patients never attempt or commit suicide, suggesting a predisposition toward suicidal behavior that is independent of the underlying depressive disorder^3^,^4^. Furthermore, genetic influences on suicidal behavior appear to be independent from the genetic susceptibility to MDD^5^, and there is no observed relationship between antidepressant or other psychotropic use and suicide attempts in depressed patients^6^. Thus, an improved understanding of the molecular basis underlying suicide predisposition should better guide the identification of biological predictors of suicide risk to improve patient outcomes.

Unfortunately, the search for clinically useful predictors for suicidal risk has been inconclusive. Demographic and psychological risk factors – such as age, gender, marital status, social and occupational functioning, comorbidities, prior suicidal behavior, and hopelessness – have yielded poor results^7^,^8^,^9^. Moreover, as most suicide risk factors have low specificities and the suicide rate is relatively low, it has been difficult to identify biomarkers for suicidal behavior^10^. The candidate biomarkers for suicidal risk that have shown promise – such as platelet monoamine oxidase activity and CSF 5-hydroxyindoleacetic acid (5-HIAA) – are neither standardized nor clinically practical^11^,^12^,^13^. Notably, dexamethasone suppression test (DST) non-suppression has also been associated with suicidal behavior but possesses low sensitivity and variable specificity^14^.

To address this challenge, several lines of evidence suggest that the peripheral circulation may be adversely affected in individuals at risk for suicide and, therefore, may serve as a source of useful biomarkers for suicidal behavior. For example, Le-Niculescu *et al*. has identified several blood-based protein biomarkers for suicide risk in bipolar patients^15^. Moreover, peripheral metabolic abnormalities in MDD patients have been associated with an increased risk of suicidal behavior^16^, and a history of attempted suicide in young adults has been shown to be a significant independent predictor of premature mortality from cardiovascular disease^17^. On the basis of this previous evidence, we hypothesized that plasma may serve as an effective biosample for assessing suicidal risk in MDD patients.

Since suicidal behavior is a multi-factorial phenomenon, a model of suicide-associated protein alterations that incorporates multiple, largely independent predictors should yield greater predictive power. To this end, proteomics – defined as the analysis of the protein complement of a cell, tissue, or organism at a certain time under precisely defined conditions^18^ – can better enable the identification of novel candidate predictors of suicidal risk. In this study, we applied a complementary plasma proteomic approach integrating 2-DE-MALDI-TOF/TOF MS and iTRAQ-LC-MS/MS platforms in order to identify differential plasma proteins from drug-naïve majorly depressed suicide attempters (MDD-SA), majorly depressed suicide non-attempters (MDD-NA), and healthy control (HC) subjects. These findings should improve our understanding of alterations to the plasma proteome that may signal heightened suicidal risk in MDD patients.

## Results

### Subject Characteristics

The detailed demographic data acquired from all recruited subjects are summarized in Table 1. The three groups did not differ on any key demographic characteristics (e.g., age, gender, or BMI). Moreover, there were no significant differences in mean HDRS scores between MDD-SA and MDD-NA subjects.

The 61 MDD-SA subjects displayed a history of a suicide attempt. In these MDD-SA subjects, the median time interval between plasma sampling and the most recent suicide attempt was 35 days (range: 0–140 days). The most common reported methods of suicide attempt (in order of frequency) were psychotropic medication overdose, ingestion of a pesticide or another chemical agent, hanging, and wrist-cutting.

### 2-DE-MALDI-TOF/TOF MS Identification of Differential Proteins

**Figure 1:**
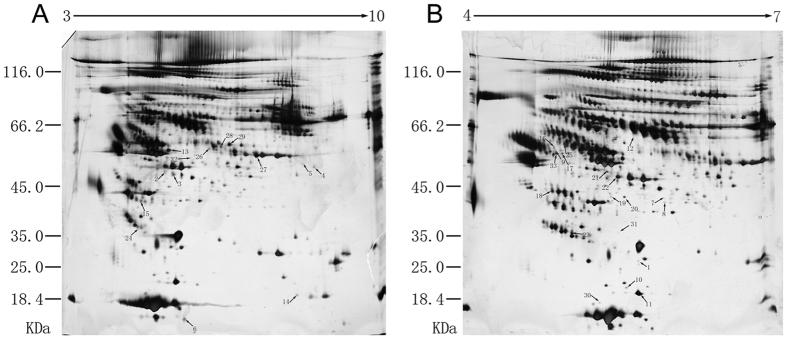
Representative Silver-Stained 2-DE Gel Images. Proteins were separated on (**A**) pH 3–10 non-linear and (**B**) pH 4–7 linear IPG strips. The second dimension was performed using 10% SDS-PAGE. Gel images were analyzed using PDQuest. The 25 unique proteins identified by MALDI-TOF/TOF MS are detailed in Supplementary Table 1. Acronyms: 2-DE, two-dimensional electrophoresis; IPG, immobilized pH gradient; SDS-PAGE, sodium dodecyl sulfate polyacrylamide gel electrophoresis; MALDI-TOF/TOF MS, matrix-assisted laser desorption/ionization time-of-flight/time-of-flight mass spectrometry.

The immunodepleted protein fraction was run on gels with pH 3–10 NL and pH 4–7 L ranges, and the obtained maps were visualized by silver staining (Fig. 1). Approximately 1100 protein spots on pH 3–10 NL range gels (Fig. 1A) and approximately 1200 protein spots on pH 4–7 L range gels (Fig. 1B) were detected. Through PDQuest analysis, 33 differential spots were identified; specifically, six spots were upregulated and 27 spots were downregulated in MDD-SA subjects as compared to MDD-NA subjects. From the foregoing 33 differential spots, MALDI-TOF/TOF MS yielded 25 unique differential proteins (Supplementary Table 1).

### iTRAQ-LC-MS/MS Identification of Differential Proteins

The identified proteins in the HC subjects were used for both MDD-SA vs. HC and MDD-NA vs. HC comparisons. The false discovery rate (FDR) calculated by searching the data against a decoy database was 1%. Only differential proteins displaying a statistically significant ±1.2 fold-change (*p* < 0.05) were selected for further analysis. These cut-offs were selected based on literature investigating the reproducibility of iTRAQ™ quantification^19^. A total of 293 differential proteins were identified in MDD-SA-based and MDD-NA-based iTRAQ-LC-MS/MS analyses. Of these, 29 and 31 were significantly differentiated in MDD-SA and MDD-NA relative to HC subjects, respectively, while 20 were significantly differentiated in both MDD groups relative to the HC group (Supplementary Table 2). No proteins were simultaneously identified by 2-DE-MALDI-TOF/TOF MS and iTRAQ-LC-MS/MS.

### Altered Biological Networks

MetaCore analysis was conducted to build biological networks and illustrate statistically plausible biological linkages between the 45 differential proteins identified above (i.e., 25 proteins from 2-DE-MALDI-TOF/TOF MS and 20 proteins from iTRAQ-LC-MS/MS; Supplementary Tables 1 and 2, respectively). The resulting top ten canonical pathway maps and GeneGo process networks are detailed in Table 2. The ‘blood coagulation’ and ‘immune response’ pathway maps displayed the largest statistical significance (*p* < 0.001). Moreover, the ‘blood coagulation’ and ‘inflammation’ process networks also showed strong statistical significance (*p* < 0.001). In order to identify the most physiologically-relevant differential proteins, we found that 32 of the 45 differential proteins mapped onto a network of relevant transcription factor linkages (data not shown), with 28 of these 32 differential proteins possessing established coagulatory and/or inflammatory functions (Supplementary Tables 1 and 2).

### Western Blotting Validation of Select Candidate Differential Proteins

**Figure 2:**
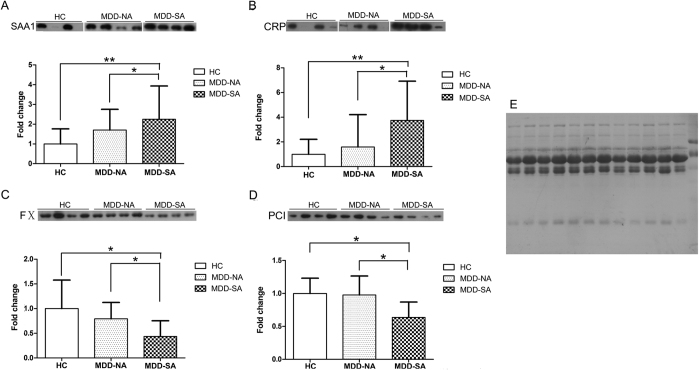
Western Blotting Validation of Differential Proteins. A total of 25 differential proteins (Supplementary Table 3) were analyzed by Western blotting for significant changes in individual MDD-SA, MDD-NA, and HC samples (n = 12 each). Only 4 of these 25 differential proteins – SAA1, CRP, FX, and PCI – displayed significant dysregulation by Western blotting. (**A**,**B**) SAA1 and CRP were significantly upregulated in MDD-SA subjects as compared to both MDD-NA and HC subjects. (**C**,**D**) FX and PCI expression were significantly downregulated in MDD-SA subjects as compared to both MDD-NA and HC subjects. (**E**) Total protein staining by Coomassie Blue employed as the loading control. A single asterisk (*) indicates significant change with *p* < 0.05, ** indicates *p* < 0.01, and *** indicates *p* = 0.000. Acronyms: MDD-SA, majorly depressed suicide attempter; MDD-NA, majorly depressed non-attempter; HC, healthy control; SAA1, serum amyloid A protein 1; CRP, C-reactive protein; FX, coagulation factor X; PCI, protein C inhibitor. Data are presented as means ± error bars representing standard deviations (SD’s).

From these 28 differential proteins with established coagulatory and/or inflammatory functions, 25 candidate differential proteins were selected for Western blotting validation based on the commercial availability of antibodies (Supplementary Table 3). Consistent with 2-DE-MALDI-TOF/TOF MS findings, only four proteins – SAA1, CRP, FX, and PCI – demonstrated significantly altered levels in MDD-SA subjects relative to both MDD-NA and HC subjects, but did not show any significant differentiation between MDD-NA and HC subjects (Fig. 2). The differential expression of the other proteins found by 2-DE-MALDI-TOF/TOF MS or iTRAQ-LC-MS/MS did not reach statistical significance (data not shown).

### ELISA Validation of Extrinsic Pathway Proteins

**Figure 3:**
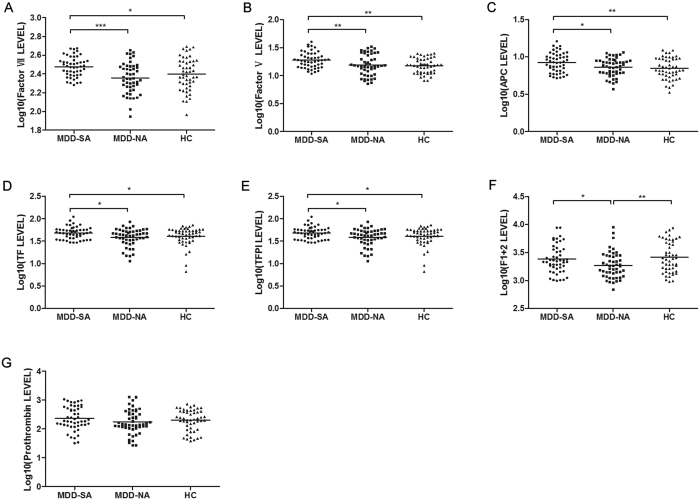
ELISA Validation of Extrinsic Pathway Proteins. Seven proteins analyzed by ELISA displayed significant changes between MDD-SA, MDD-NA, and HC samples (n = 49 each). (**A**) FVII, (**B**) FV, (**C**) APC, (**D**) soluble TF, and (**E**) TFPI were significantly upregulated in MDD-SA subjects compared to both MDD-NA and HC subjects. (**F**) F1 + 2 was significantly upregulated in MDD-SA compared to MDD-NA subjects and downregulated in MDD-SA compared to HC. (**G**) There was no significant changes in prothrombin among the three groups. A single asterisk (*) indicates significant change with *p* < 0.05, ** indicates *p* < 0.01, and *** indicates *p* = 0.000. Acronyms: ELISA, enzyme-linked immunosorbent assay; MDD-SA, majorly depressed suicide attempter; MDD-NA, majorly depressed non-attempter; HC, healthy control; TF, tissue factor; TFPI, tissue factor pathway inhibitor; FVII; coagulation factor FVII; APC, activated protein C; F1 + 2, prothrombin fragment 1 + 2.

Expression of seven extrinsic pathway proteins – soluble TF, TFPI, FVII, APC, FV, prothrombin, and F1 + 2 – were assessed using individual samples from the MDD-SA, MDD-NA, and HC subjects containing both drug-naïve and antidepressant-treated MDD subjects. FVII, APC, FV, TF, and TFPI were all significantly increased in MDD-SA subjects relative to both MDD-NA and HC subjects with no significant differences observed between MDD-NA and HC subjects (Fig. 3A–E). F1 + 2 was significantly increased in MDD-SA subjects relative to MDD-NA but decreased relative to HC subjects (Fig. 3F). There were no significant differences in prothrombin among the three groups (Fig. 3G).

### Heparin Therapy Promotes FST Immobility in Rats

**Figure 4:**
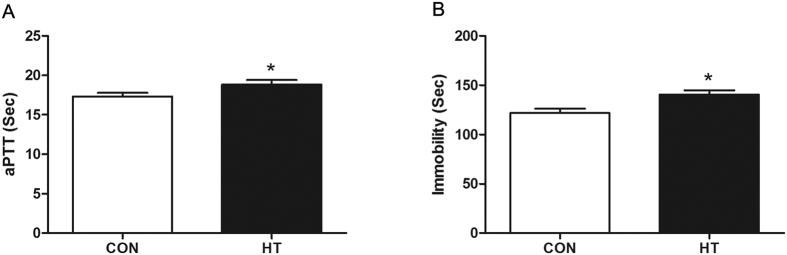
Anticoagulant Activity and Forced Swimming Test (FST) Immobility Post-Heparin Administration. (**A**) The effects of heparin on plasma clotting times (aPTT). (**B**) Immobility times in the forced swimming test (FST) between control (n = 9) and heparin-treated rats (n = 29). An asterisk (*) indicates significant change (*p* < 0.05). Acronyms: aPTT, activated partial thromboplastin time; CON, control group; HT, heparin-treated group. Data are presented as means ± error bars representing standard deviations (SD’s).

Based on the foregoing findings, we hypothesized that heparin treatment – which inactivates the extrinsic pathway proteins thrombin, FXa, and FIXa^20^ – may promote depression-like behavior in rodents as measured by increased passive (immobility) behavior in the FST. Following seven days of heparin (or control saline) administration, activated partial thromboplastin time (aPTT) levels were measured in rat plasma samples to validate effective heparinization in heparin-treated rats. As expected, aPTT levels were significantly elevated in the heparin-treated subjects as compared to saline-treated control subjects (*p* = 0.0237; Fig. 4A). FST immobility time was significantly elevated in heparin-treated subjects as compared to saline-treated control subjects (*p* = 0.0295; Fig. 4B).

## Discussion

This study is the first reported proteomic investigation of plasma-based biological predictors of suicidal behavior in MDD patients. After Western blotting validation of 25 differential proteins identified by 2-DE-MALDI-TOF/TOF MS and iTRAQ-LC-MS/MS analyses, four candidate predictors – CRP, SAA1, FX, and PCI – were significantly differentiated in MDD-SA subjects relative to both MDD-NA and HC subjects. Based on these Western blotting findings, we hypothesized that the predisposition to suicide in MDD patients was associated with perturbation of the extrinsic pathway. Therefore, ELISA validation was conducted on seven additional extrinsic pathway proteins, which showed that six more candidate predictors – TF, FVII, FV, TFPI, APC, and F1 + 2 – were significantly differentiated in MDD-SA subjects relative to both MDD-NA and HC subjects. As these ELISA findings were based on 147 samples (n = 49 in each group) containing both drug-naïve and antidepressant-treated MDD subjects, these candidate predictors demonstrate promise in distinguishing suicide risk in a realistic MDD patient population consisting of both medicated and non-medicated depressed individuals.

Through Metacore analysis of 2-DE-MALDI-TOF/TOF MS and iTRAQ-LC-MS/MS findings (Table 2, Supplementary Tables 1 and 2), two biologic processes – coagulation and inflammation – were primarily associated with suicidal behavior in MDD. Consistent with these findings, extensive molecular crosstalk between inflammatory and coagulatory processes has been well-established. Specifically, inflammatory cytokines increase cell-bound TF expression on monocytes and induce endothelial cells to release soluble TF while downregulating APC production^21^,^22^. Increased inflammatory cytokine levels have been well-established in depressed patients^23^,^24^. However, there have been conflicting conclusions regarding the relationship between coagulation and depression^25^,^26^,^27^,^28^,^29^.

Although Le-Niculescu *et al*.’s recent bioinformatic analyses revealed stress, inflammation, and apoptosis as biological pathways associated with suicidality in bipolar disorder^15^, this study is the first to investigate the relationships between inflammation, coagulation, and suicidal behavior in MDD. As discussed below, two inflammatory proteins (CRP, SAA1) and eight coagulatory proteins (TF, FVII, FX, FV, TFPI, APC, PCI, F1 + 2) demonstrated significantly differentiated expression in MDD-SA subjects relative to both MDD-NA and HC subjects (Figs 2 and 3).

CRP, a liver-derived pentraxin secreted in response to inflammatory cytokines that activates the complement system^30^, was increased in MDD-SA relative to MDD-NA and HC subjects. Elevated CRP has been established in depressed patients by two meta-analyses^24^,^31^. SAA1, the predominant apolipoprotein isoform of high density lipoprotein (HDL)^32^, was also increased in MDD-SA relative to MDD-NA and HC subjects. Both CRP and SAA1 are liver-derived acute-phase reactants that are upregulated in response to inflammatory cytokines and are also centrally involved in inflammation-coagulation crosstalk by inducing monocytes to express cell-bound TF^33^,^34^.

**Figure 5:**
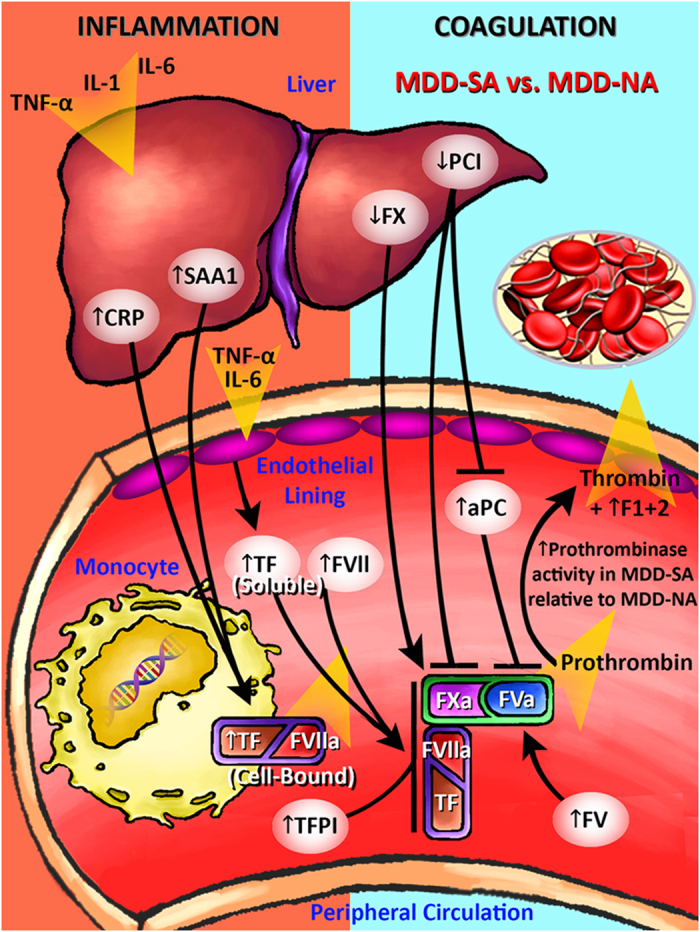
Inflammation-Coagulation Crosstalk and Extrinsic Pathway Activation in Majorly Depressed Suicide Attempters. Inflammatory cytokines induce endothelial cells to express soluble TF. More potent upregulation of CRP and SAA1 in MDD-SA subjects induces increased monocytic expression of cell-bound TF, which catalyzes the conversion of increased soluble TF and circulating FVII to generate the TF-FVIIa complex that efficiently converts the increased FX to FXa. FXa and thrombin then mediate the activation of increased FV to form FVa, which, in turn, binds to FXa to form prothrombinase (FVa-FXa complex). TFPI upregulation indicates inhibition of the TF-FVIIa complex and FXa. PCI downregulation indicates disinhibition of FXa. APC upregulation is likely a result of PCI downregulation. The cumulative effect of these differential changes in protein expression yields increased relative prothombinase activity in MDD-SA relative to MDD-NA subjects (Fig. 6). Acronyms: MDD, major depressive disorder; MDD-SA, majorly depressed suicide attempter; MDD-NA, majorly depressed non-attempter; HC, healthy control; TNF-alpha, tumor necrosis factor-alpha; IL-1, interleukin-1; IL-6, interleukin-6; CRP, C-reactive protein; SAA1, serum amyloid A protein 1; TF, tissue factor; FVII, coagulation factor FVII; FVIIa, activated coagulation factor FVII; FX, coagulation factor X; FXa, activated coagulation factor X; FV, coagulation factor V; FVa, activated coagulation factor V; TFPI, tissue factor pathway inhibitor; PCI, protein C inhibitor; APC, activated protein C.

Soluble TF, FVII, FX, and FV – all protein constituents of the coagulation cascade (Fig. 5) – were shown to be increased in MDD-SA relative to MDD-NA and HC subjects. At the initiation of the extrinsic pathway, cell-bound TF on monocytes catalyze the generation of the TF-FVIIa complex from endothelium-released soluble TF and circulating FVII. This complex efficiently converts FX to FXa. Then, FXa and thrombin mediate the activation of FV to form FVa, which, in turn, binds to FXa to form prothrombinase (FVa-FXa complex), which promotes coagulation by cleaving prothrombin to generate thrombin^35^,^36^.

In addition, three regulators (TFPI, APC, and PCI) and one by-product (F1 + 2) of the coagulation cascade were dysregulated in MDD-SA relative to MDD-NA and HC subjects. TFPI, a potent inhibitor of coagulation that was significantly upregulated in MDD-SA relative to MDD-NA and HC subjects, binds to FXa and the TF-FVIIa complex, forming an inactive quaternary complex TF-FVIIa-TFPI-FXa^37^. APC, another inhibitor of coagulation that was significantly increased in MDD-SA relative to MDD-NA and HC subjects, cleaves and inhibits FVa^38^. PCI, another inhibitor of coagulation that was significantly decreased in MDD-SA relative to MDD-NA and HC subjects, inhibits FXa and is the primary inhibitor of APC in human plasma^39^. Finally, F1 + 2 – the byproduct of prothrombin’s conversion to thrombin that is considered to be a reliable marker of the circulating thrombin levels^40^ – was significantly upregulated in MDD-SA compared to MDD-NA subjects and downregulated in MDD-SA compared to HC.

**Figure 6:**
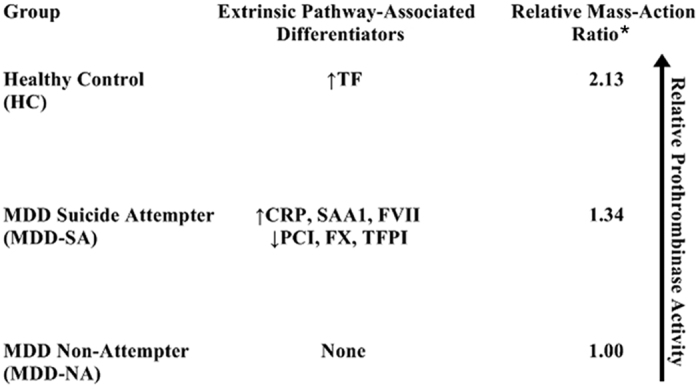
Extrinsic Pathway Differentiators and Relative Prothrombinase Activity. Based on the mean values of F1 + 2 and prothrombin from the three groups, HC subjects displayed higher relative prothrombinase activity than both MDD-SA and MDD-NA subjects, and MDD-SA subjects displayed a higher relative prothrombinase activity than MDD-NA subjects. ^*^The relative mass-action ratio applied here, Γr = [F1 + 2]^2^/[prothrombin]_x_)/([F1 + 2]^2^_MDD-NA_/[prothrombin]_MDD-NA_, was derived from the prothrombinase reaction (i.e., prothrombin → F1 + 2 + thrombin) with a 1:1 molar equivalency of the F1 + 2 and thrombin products, leading to the [F1 + 2]-squared term. As opposed to simply comparing F1 + 2 levels, this ratio was applied as a more accurate metric of relative prothrombinase activity to control for intergroup variations in prothrombin levels. Acronyms: MDD-SA, majorly depressed suicide attempter; MDD-NA, majorly depressed non-attempter; HC, healthy control; F1 + 2, prothrombin fragment 1 + 2.

Therefore, MDD, independent of suicidality, is associated with a proinflammatory state as evidenced by significantly higher CRP and SAA1 levels in both MDD-SA and MDD-NA relative to HC subjects (Fig. 2) accompanied by a hypothrombotic state as evidenced by significantly lower F1 + 2 levels and lower relative prothrombinase activity in MDD-SA and MDD-NA relative to HC subjects (Figs 2 and 6).

In contrast, suicidal behavior in MDD is associated with a more pronounced inflammatory phenotype, as evidenced by significantly higher CRP and SAA1 levels (Fig. 2) and a more pronounced prothrombotic phenotype as evidenced by higher relative prothrombinase activity (Fig. 6) and significantly increased levels of soluble TF, FVII (~1% of FVII circulates as FVIIa), FX, FV, and F1 + 2 (Fig. 3)^41^. TFPI upregulation indicates inhibition of the TF-FVIIa complex and FXa, while PCI downregulation indicates disinhibition of FXa (Figs 3 and 5). APC upregulation is likely a result of the downregulation of PCI^39^. These findings may also explain conflicting previous studies regarding the expression of coagulative factors in depressed patients^25^,^26^,^27^,^28^,^29^, as prior studies failed to selectively enroll or analytically distinguish depressed suicidal attempters and non-attempters.

As the foregoing findings reveal a hypothrombotic state in MDD-SA and MDD-NA subjects (Figs 2 and 6), we hypothesized that heparin treatment – which induces a hypothrombotic state through inactivating the extrinsic pathway proteins thrombin, FXa, and FIXa^20^ – may promote depression-like behavior in rodents. Depression and suicidal behavior cannot be directly modeled in rodents, but component traits associated with these conditions can be investigated^42^. Despair represents one component trait associated with both depression and suicidal behavior that can be measured in rodent models through increased passive (immobility) behavior during the forced swimming test. Consistent with our hypothesis, we found that seven days of effective heparin treatment – which induced a hypothrombotic state as measured by an elevated aPTT – did significantly increase FST immobility time (Fig. 4). Unfortunately, we did not measure the dose-dependence relationship (if any) between heparin dosing and passive (immobility) behavior nor did we assess the effects of heparin treatment on individual extrinsic coagulation pathway proteins. Future research in this field should examine these questions to better define the relationship between hypothrombotic states, extrinsic pathway dysregulation, and depression-like behavior.

There are several limitations to this study that should be addressed here. First, there may be a possibility that self-wounding at the time of suicide attempt may have affected the expression of coagulation molecules in MDD-SA subjects. However, previous research has demonstrated that the initial hemostatic response to wounding (i.e., a process that releases clotting factors resulting in platelet aggregation, activation of the coagulation pathway, and clot formation) is very short in duration^43^ and that the degree of coagulation is not statistically significant for wound contraction^44^. As self-wounding was one of the least common methods of suicide attempt and the median time interval between plasma sampling and the most recent suicide attempt was 35 days (range: 0–140 days), we can reasonably surmise that the changes to extrinsic pathway protein expression observed in MDD-SA subjects were likely not significantly influenced by self-wounding. Second, there may be a possibility that lowered physical activity levels during hospitalization may have affected the expression of coagulation molecules. However, it is well-established that extrinsic pathway activation is not significantly influenced by physical activity levels^45^. Therefore, our findings cannot be reasonably attributed to lowered physical activity levels during hospitalization. Third, the present study does not establish any causal relationships between extrinsic pathway dysregulation and suicidal tendency in MDD patients. However, this study does provide data supporting a significant association between extrinsic pathway dysregulation and suicidal tendency in MDD patients as a platform for future research.

In conclusion, depressed suicide attempters occupy a phenotypic “middle ground” between hypothrombotic depressed non-attempters and euthrombotic healthy individuals (Fig. 6) accompanied by extrinsic pathway activation through differential expression of ten extrinsic pathway-associated proteins (↑CRP, ↑SAA1, ↑TF, ↑FVII, ↑FV, ↑TFPI, ↑APC, ↑F1 + 2; ↓FX, ↓PCI), revealing an extrinsic pathway biomarker that can be applied in predicting and monitoring suicidal risk in MDD patients (Fig. 5). As SSRI antidepressants may collaterally perturb extrinsic pathway factors^46^,^47^, changes in extrinsic pathway proteins may be associated with the increased risk of suicidal behavior observed in depressed patients during the first month of SSRI therapy^48^. As an interesting tangent for future investigation, our previously published metabolomic findings have also shown that suicide attempters occupy a phenotypic “middle ground” with respect to plasma levels of alanine, glycine, and glucose^16^. Thus, suicidal MDD may possess a distinct phenotype entirely distinguishable from non-suicidal MDD. Future “omics”-based research should aim to better differentiate suicidal MDD from non-suicidal MDD, as better defining this phenotypic distinction can have significant ramifications on the diagnosis, monitoring, and treatment of MDD patients.

## Methods and Materials

### Ethics Statement

The human and animal experimental protocols of this study were approved by the Ethics Committee of Chongqing Medical University (Chongqing, China). All protocols were performed in accordance with relevant guidelines and regulations. Written informed consent was obtained from all human participants after complete description of the study. Animal care and treatment were conducted in accordance with the National Institute of Health’s Guide for the Care and Use of Laboratory Animals (NIH Publications No. 80-23, revised 1996).

### Subject Recruitment

Sixty-one MDD subjects who attempted suicide during the MDD episode (MDD-SA, suicide attempters) and sixty-one MDD subjects who have never attempted suicide (MDD-NA, non-attempters) were recruited in the psychiatric department of the First Affiliated Hospital of Chongqing Medical University (Chongqing, China). A ‘suicide attempt’ was defined as a demonstrable self-harming behavior with intent to terminate one’s own life. A suicide attempt ranged from high-potential suicide attempts (demonstrating high intention and planning where survival was fortuitous) to low-potential suicide attempts (characterized by poorly-planned, impulsive attempts triggered by a social crisis, ambivalence, and demonstrating a strong element of an appeal for help)^49^. HC subjects were recruited from the medical examination center at Chongqing Medical University. Inclusion and exclusion criteria for MDD and HC subjects were as previously described^50^.

### Plasma Sample Collection and Preparation

Blood samples (5 ml) were collected in EDTA-vacutainers (BD vacutainers catalog #367863) by venipuncture between 8:00–10:00 a.m., immediately placed on ice, and centrifuged at 3000 rpm for 15 min at 4 °C. The resultant plasma was aliquoted and stored at −80 °C within 1 h of collection. Pooled plasma samples were generated by combining equal volumes of the 12 individual plasma samples from each of the three groups (i.e., drug-naïve MDD-SA, drug-naïve MDD-NA, and HC candidates). According to the manufacturer’s instructions, a 420 μl volume from every pool was depleted of the most abundant proteins with a MARS-human 7 HPLC column for 2-DE-MALDI-TOF/TOF MS and MARS-human 14 HPLC column for iTRAQ (Agilent, Santa Clara, California).

### Protein Identification by 2-DE-MALDI-TOF/TOF MS

Processed sample pools were purified using TCA precipitation, then air-dried for five minutes. The protein was dissolved in a dissociation solution (7 mM urea, 2 M thiourea, 4% CHAPS, 50 mM DTT, 0.2% 3–10 Bio-Lyte, Bio-rad) and measured using the Bradford method. Immediately prior to IEF, the samples were further diluted to 100 μg/350 μl with dissociation solution. Twelve gels were developed, as every pooled sample was run four times to control for gel variation, and imaged as previously described^51^. The MS integrated with MS/MS spectra were searched against the International Protein Index (IPI Human v3.78, 86392 entries) using GPS Explorer version 3.78 (Applied Biosystems) and MASCOT version 2.1 (Matrix Science). The search parameters were set as previously described^51^.

### Protein Digestion and iTRAQ Labeling

Each sample consisted of a pool of plasma from 12 HC, 12 MDD-NA, or 12 MDD-SA individuals. The proteins in each sample were denatured, reduced, alkylated, and digested with sequencing-grade modified trypsin with a protein-to-enzyme ratio of 20:1 at 37 °C overnight and then labeled with the following iTRAQ reagent tags in duplicate: 113 and 118 for HC, 114 and 119 for MDD-NA, and 115 and 121 for MDD-SA subjects.

### LC-MS/MS Analysis by Q Exactive

iTRAQ-labeled peptides were mixed and fractionated by SCX chromatography using the AKTA Purifier system (GE Healthcare), then 10 μl of each fraction was injected for LC-MS/MS analysis using Q Exactive mass spectrometer that was coupled to an Easy-nLC (Thermo Fisher Scientific). The peptide mixture (5 μg) was loaded into a C18-reversed phase column (15 cm long, 75 μm inner diameter), packed in-house with RP-C18 5 μm resin in buffer A (0.1% formic acid), and separated with a linear gradient of buffer B (80% acetonitrile, 0.1% formic acid) at a flow rate of 250 nl/min controlled by IntelliFlow over 140 min. Survey scans were acquired at a resolution of 70000 at m/z 200 and resolution for HCD spectra was set to 17500 at m/z 200. Each SCX fraction was analyzed in duplicate.

### Sequence Database Search and Data Analysis

MS/MS spectra were searched using MASCOT engine (Matrix Science, London, UK; version 2.2) against the IPI human sequence database v3.87. For protein identification, the following options were used: peptide mass tolerance, 20 ppm; MS/MS tolerance, 0.1 Da; enzyme, trypsin; missed cleavage, 2; fixed modification, iTRAQ8plex (K), iTRAQ8plex (N-term); variable modification, oxidation (M); and decoy database pattern, reverse, and FDR < 0.01. The MASCOT search results for each SCX elution were further processed using the ProteomicsTools (v.3.05), which includes the programs BuildSummary, Isobaric Labeling Multiple File Distiller, and Identified Protein iTRAQ Statistic Builder (Research Center for Proteome Analysis, http://www.proteomics.ac.cn/)^52^.

### Biological Network Analysis Using MetaCore

As previously described^50^, the gene symbols of differential proteins were uploaded into MetaCore version 6.6 (GeneGo) for biological network construction.

### Western Blotting Validation of Differential Proteins

Thirty-six individual plasma samples – consisting of HC, MDD-SA, and MDD-NA subjects (n = 12 each) – were employed in the Western blotting validation. In contrast to the 2-DE-MALDI-TOF/TOF MS and iTRAQ-LC-MS/MS analyses, individual crude plasma samples were used for this validation. Plasma proteins (5 μg per sample) were separated by SDS-PAGE. After electrophoresis, the proteins were electrotransferred onto PVDF membranes (Millipore). After blocking in 5% skim milk in TBST, the membranes were incubated overnight at 4 °C with the primary antibodies. All membranes were washed and incubated with their respective horseradish peroxidase-coupled secondary antibody (Bio-Rad). After extensive washing, protein bands detected by the antibodies were visualized by the enhanced chemiluminescence method and exposed to autoradiography film. After immunodetection, the membranes were stained with Coomassie Blue as an internal control for normalization^53^. The Western blot signals were densitometrically quantified in each sample with Quantity One software (Bio-Rad).

### ELISA Validation of Extrinsic Pathway Proteins

A total of 147 individual plasma samples – consisting of HC, MDD-SA, and MDD-NA subjects (n = 49 each) containing both drug-naïve and antidepressant-treated MDD subjects – were employed in the ELISA validation. Seven extrinsic pathway proteins were analyzed using commercially available kits following the manufacturers’ directions: soluble (non-cell bound) tissue factor (TF) (R&D Systems, Minneapolis, MN, USA), tissue factor pathway inhibitor (TFPI) (R&D Systems, Minneapolis, MN, USA), factor VII (FVII) (Abcam, Cambridge, UK), activated protein C (APC) (Life Science, Wuhan, China), coagulation factor V (FV) (Innovative Research, Plymouth, USA), prothrombin (Abcam, Cambridge, UK), and prothrombin fragment 1 + 2 (F1 + 2) (Life Science, Wuhan, China). No more than two freeze-thaw cycles were allowed per specimen. All samples were analyzed in duplicate.

### Heparin Administration in Rats

Healthy male Sprague-Dawley rats were obtained from the Animal Center at Chongqing Medical University. Rats were individually housed and acclimatized to the animal colony for one week before initiation of experimentation. All rats received a standard rodent diet and tap water ad libitum under a 12 h light–dark cycle (lit from 7:30 to 19:30), a temperature of 21–22 °C, and humidity of 55 ± 5%.

After a seven-day adaptation to these standard conditions, the animals were randomly segregated into two groups: a heparin-treated group (n = 29) and a control group (n = 9). Animal weight was approximately 200 g at the start of pharmacological treatment. Heparin (5 g/l body weight in saline, Solarbio) was injected intraperitoneally once daily for seven days. Control mice were injected with an equivalent amount of saline.

### Post-Heparin aPTT

After seven days of heparin (or control saline) administration, blood was collected from the angular vein into plastic tubes (2.0 ml) containing 1/10 volume of 3.8% trisodium citrate dehydrate (Junnuo, Shandong, China) at the end of drug infusion. Plasma was obtained by centrifugation at 1500 g for 10 minutes at 4 °C, and aPTT was analyzed by a Sysmex CA-7000 Coagulation Analyzer (Sysmex, Kobe, Japan)^54^.

### Post-Heparin FST

After seven days of heparin (or control saline) administration, the FST was performed as described previously^55^. The rats were placed individually in Plexiglas cylinders (40 cm in height, 20 cm in diameter) filled with water (24 ± 1 °C) up to a height of 30 cm. A 15-min pretest period was followed 24 h later by a 5-min test period during which the total immobility time was recorded. The test was monitored by a video surveillance system (SMART, Panlab SL, Barcelona, Spain). Water in the cylinders was changed before each trial.

### Statistical Analysis

Statistical analysis was performed using the Statistical Package of Social Science (SPSS) for Windows version 19.0. All data were expressed as means ± standard deviations (SD’s) unless otherwise noted. The analysis of variance (ANOVA) test was applied to identify proteins with significant expression differences across the three groups. Statistical significance was set at *p* < 0.05.

## Additional Information

**How to cite this article**: Yang, Y. *et al*. The Extrinsic Coagulation Pathway: a Biomarker for Suicidal Behavior in Major Depressive Disorder. *Sci. Rep.* **6**, 32882; doi: 10.1038/srep32882 (2016).

## Supplementary Material

Supplementary Information

## Figures and Tables

**Table 1:** Demographic and Clinical Features of Recruited Subjects.

Variable	MDD-SA	MDD-NA	HC	MDD-SA/MDD-NA	MDD-SA/HC
				*P*-value^a^	*P*-value^a^
Proteomic Analysis and Western Blot Validation					
Sample size	12	12	12	—	—
Sex (M/F)	3/9	3/9	3/9	1.000	1.000
Age (year)^b^	28.00 ± 12.03	35.83 ± 11.24	31.00 ± 12.77	0.120	0.545
BMI^b^	21.6 ± 2.4	22.7 ± 1.9	21.5 ± 2.0	0.510	0.405
HAM-D scores^b^	22.58 ± 5.07	23.08 ± 3.97	—	0.790	—
ELISA Validation					
Sample size	49	49	49	—	—
Sex (M/F)	18/31	15/34	16/33	0.525	0.672
Age (year)^b^	33.67 ± 12.17	36.29 ± 11.19	33.13 ± 5.79	0.216	0.810
BMI^b^	22.3 ± 3.6	21.3 ± 2.0	21.9 ± 4.2	0.490	0.872
HAM-D scores^b^	26.14	24.94	—	0.291	

^a^Two-tailed student *t*-test for continuous variables (age, BMI, and HDRS Scores); Chi-square analyses for categorical variables (sex).

^b^Age, BMI, and HAM-D scores are presented as means ± SD’s.

Abbreviations: HC, healthy controls; MDD-SA, majorly depressed suicide attempters; MDD-NA, majorly depressed suicide non-attempters; M, male; F, female; BMI, body mass index; HAM-D, Hamilton Depression Rating Scale.

**Table 2:** Top Ten-Ranking GeneGo Pathway Maps and GeneGo Process Networks Associated with the Differential Proteins Based on MetaCore Database Analysis.

	*P*-value
GeneGo Pathway Maps	
1. Blood coagulation	1.444 × 10^−13^
2. Immune response: classical complement pathway	4.388 × 10^−7^
3. Immune response: alternative complement pathway	2.215 × 10^−4^
4. Immune response: lectin-induced complement pathway	4.376 × 10^−4^
5. Immune response: sialic-acid receptors (Siglecs) signaling	3.643 × 10^−2^
6. Ascorbate metabolism	5.417 × 10^−2^
7. Development: thrombopoetin signaling via JAK-STAT pathway	6.582 × 10^−2^
8. Glycolysis and gluconeogenesis p.3/Human version	7.159 × 10^−2^
9. Glycolysis and gluconeogenesis p.3	7.159 × 10^−2^
10. Development: leptin signaling via JAK/STAT and MAPK cascades	7.446 × 10^−2^
GeneGo Process Networks	
1. Blood coagulation	9.927 × 10^−11^
2. Inflammation: kallikrein-kinin system	3.569 × 10^−10^
3. Inflammation: complement system	1.759 × 10^−7^
4. Inflammation: IL-6 signaling	3.438 × 10^−7^
5. Signal transduction: leptin signaling	3.322 × 10^−3^
6. Cell adhesion: platelet-endothelium-leucocyte interactions	3.399 × 10^−3^
7. Development: ossification and bone remodeling	1.313 × 10^−2^
8. Proteolysis: ECM remodeling	1.336 × 10^−2^
9. Inflammation: protein C signaling	2.517 × 10^−2^
10. Development: angiogenesis regulation	4.098 × 10^−2^
