# Supplementary material for: The Extrinsic Coagulation Pathway: a Biomarker for Suicidal Behavior in Major Depressive Disorder
Source: Sci Rep. 2016 Sep 8;6:32882. doi: 10.1038/srep32882 (PMC5015115; doi:10.1038/srep32882)
Supplement: Supplementary Information [file srep32882-s1.doc]

**Supplementary Information**

**The Extrinsic Coagulation Pathway: a Biomarker for Suicidal Behavior in Major Depressive Disorder**

Yongtao Yang ^1,2,3*^, Jin Chen ^1,2,3*^, Chengyu Liu ^1,2*^, Liang Fang ^2,3,4*^, Zhao Liu ^2,3*^, Jing Guo ^1,2,3^, Ke Cheng ^1,2,3^ , Chanjuan Zhou ^2,3^, Yuan Zhan ^2,3^, Narayan D Melgiri ^2,3^, Liang Zhang ^1,2,3^, Jiaju Zhong ^2,3,4^, Jianjun Chen ^2,3^, Chenglong Rao ^2,3^, & Peng Xie ^1,2,3,4^

^1^Department of Neurology, the First Afﬁliated Hospital of Chongqing Medical University, Chongqing, China

^2^Chongqing Key Laboratory of Neurobiology, Chongqing, China

^3^Institute of Neuroscience and the Collaborative Innovation Center for Brain Science, Chongqing Medical University, Chongqing, China

^4^Department of Neurology, Yongchuan Hospital of Chongqing Medical University, Chongqing, China

* These authors contributed equally to this work

**Correspondence and requests for materials should be addressed to:**

Professor Peng Xie

Department of Neurology

The First Affiliated Hospital, Chongqing Medical University

1 Yixueyuan Road, Yuzhong District, Chongqing, 400016, China

Tel: +86-23-68485490

Fax: +86-23-68485111

E-mail: *xiepeng@cqmu.edu.cn*

**Supplementary Table 1. The 25 Differential Proteins Identified by 2-DE Coupled with MALDI-TOF/TOF MS**

| **Accession**  **No. (IPI)** | **Gene Symbol** | **Protein Name** | **Protein MW**  **(Da)** | **Protein**  **PI** | **Biological Function** | **Spot**  **No.** | **Fold Change** | |
| --- | --- | --- | --- | --- | --- | --- | --- | --- |
|  |  |  |  |  |  |  | **MDD-SA/ HC** | **MDD-SA/ MDD-NA** |
| IPI00004373 | MBL2 | ★Mannose-binding protein | 26143.53 | 5.1405 | Immune response/inflammation: complement activation | 1 | +1.54 | +1.69 |
| IPI00006114 | SERPINF1 | Pigment epithelium-derived factor | 46312.21 | 6.3276 | Cell proliferation, lipid metabolism, inflammation | 2  3 | -2.08  -1.67 | -2.27  -1.70 |
| IPI00007221 | SERPINA5 | ★Plasma serine protease inhibitor | 45701.7 | 9.7593 | Blood coagulation | 4  5 | -2.86  -2.22 | -3.60  -2.96 |
| IPI00009028 | CLEC3B | ★Tetranectin | 22566.84 | 5.3639 | Blood coagulation: fibrinolysis | 6 | -2.5 | -1.88 |
| IPI00011264 | CFHR1 | Complement factor H related protein 1 | 37650.56 | 7.4243 | Immune response/inflammation: complement activation | 7  8 | -1.92  -1.72 | -1.52  -1.28 |
| IPI00019576 | F10 | ★Coagulation factor X | 54731.69 | 5.7401 | Blood coagulation | 9 | -3.23 | -2.61 |
| IPI00022391 | APCS | ★Serum amyloid P component | 25387.13 | 6.5188 | Inflammation: acute-phase response | 10  11 | -6.67  -1.82 | -3.27  -1.51 |
| IPI00022488 | HPX | Hemopexin | 51676.37 | 7.0195 | Inflammation: immunoglobulin production | 12 | +1.75 | +1.42 |
| IPI00032179 | SERPINC1 | ★Antithrombin-III | 52602.44 | 6.68 | Blood coagulation | 13 | -2.78 | -2.81 |
| IPI00165972 | CFD | Complement factor D preproprotein | 27779.73 | 7.2707 | Immune response/inflammation: complement activation | 14 | -2.86 | -2.20 |
| IPI00166729 | AZGP1 | ★Zinc-alpha-2-glycoprotein | 34258.72 | 5.9243 | Immune response/inflammation | 15 | -2.13 | -1.77 |
| IPI00215894 | KNG1 | ★Isoform LMW of kininogen-1 | 47883.21 | 6.6273 | Blood coagulation: platelet degranulation | 16  17 | +2.26  +2.38 | +1.87  +2.53 |
| IPI00218732 | PON1 | ★Serum paraoxonase | 39731.31 | 4.8869 | Lipid metabolism | 18 | -1.89 | -1.64 |
| IPI00219583 | SHBG | ★Sex hormone-binding globulin isoform 3 precursor | 32363.04 | 6.176 | Primary spermatocyte growth | 19  20 | -1.92  -1.92 | -1.85  -2.13 |
| IPI00219713 | FGG | Isoform gamma a of fibrinogen gamma chain | 49496.54 | 5.974 | Blood coagulation: platelet degranulation | 21  22 | -1.82  -3.03 | -1.65  -1.70 |
| IPI00291262 | CLU | Isoform 1 of clusterin | 52494.58 | 6.2083 | Inflammation, lipid and hormone transport | 23  24 | -2.00  -2.50 | -2.56  -2.23 |
| IPI00292946 | SERPINA7 | ★Thyroxine-binding globulin | 46324.50 | 6.2722 | Thyroid hormone-binding | 25 | -2.94 | -1.53 |
| IPI00296165 | C1R | CDNA FLJ54471, Highly similar to complement C1R subcomponent | 81889.52 | 6.3268 | Immune response/inflammation | 26 | -2.04 | -1.76 |
| IPI00298828 | APOH | β-2-glycoprotein 1 | 38298.16 | 8.0275 | Triglyceride metabolism | 27 | -1.96 | -1.94 |
| IPI00328609 | SERPINA4 | Kallistatin | 48541.97 | 7.853 | Blood coagulation | 28 | -1.89 | -1.55 |
| IPI00478003 | A2M | Alpha-2-macroglobulin | 163291.00 | 6.4306 | Immune response/inflammation: complement regulation | 29 | -1.85 | -2.07 |
| IPI00645213 | APOM | Apolipoprotein M, isoform CRA_A | 13050.94 | 7.8673 | Cholesterol efflux | 30 | +3.89 | +6.08 |
| IPI00796990 | CFI | CDNA FLJ58124, high similar to complement factor 1 | 66695.31 | 7.5365 | Immune response/inflammation: complement activation | 31 | -1.61 | -1.27 |
| IPI00935408 | CFI | ★CFI PROTEIN. | 42432.18 | 8.1267 | Immune response/inflammation: complement activation | 32 | +2.84 | +1.52 |
| IPI00953689 | AHSG | ★Alpha-2-HS-glycoprotein | 39324.68 | 5.5227 | Skeletal system development | 33 | -2.38 | -2.43 |

★ Proteins validated by Western blotting.

**Supplementary Table 2. The 20 Differential Proteins Identified by iTRAQ-**LC-MS/MS

| **Accession**  **No. IPI** | **Protein Name** | **Pep**  **Count** | **Unique Pep Count** | **Cover %** | **Biological Function** | **Theoretical MM/pI** | **Fold-Change** | |
| --- | --- | --- | --- | --- | --- | --- | --- | --- |
|  |  |  |  |  |  |  | **MDD-SA/ HC** | **MDD-SA/ MDD-NA** |
| IPI00641737 | Haptoglobin | 22 | 10 | 24.88% | Inflammation: acute-phase | 45204.78/6.13 | +1.56 | +1.79 |
| IPI00171678 | ★Dopamine beta-hydroxylase | 10 | 16 | 12.32% | Catecholamine biosynthesis | 69064.2/5.97 | -1.35 | -1.28 |
| IPI00021855 | ★Apolipoprotein C-I | 13 | 3 | 30.12% | Lipid transport | 9331.81/8.01 | -1.32 | -1.41 |
| IPI00796379 | Beta-2-microglobulin | 8 | 3 | 22.13% | Immune response/ inflammation: MHC Class I/CD8 regulation | 13945.75/6.9 | -1.36 | -1.35 |
| IPI00022389 | ★C-reactive protein | 6 | 3 | 13.84% | Inflammation: acute-phase | 25038.26/5.45 | +2.78 | +2.14 |
| IPI00552578 | ★Serum amyloid A-1 protein | 3 | 3 | 35.25% | Inflammation: acute-phase | 13531.86/6.28 | +2.01 | +1.68 |
| IPI00967086 | Hepatocyte growth factor activator short chain | 9 | 2 | 4.53% | Inflammation: macrophage stimulation | 71481.92/6.69 | -1.62 | -1.39 |
| IPI00964994 | Hyaluronan-binding protein 2 50 kDa heavy chain | 6 | 2 | 3.93% | Inflammation: vascular barrier regulation | 59863.65/5.99 | -1.40 | -1.40 |
| IPI00022731 | ★Apolipoprotein C-IV | 5 | 2 | 14.96% | Lipid transport | 14552.9/9.19 | -1.50 | -1.31 |
| IPI00027462 | ★Protein S100-A9 | 4 | 2 | 24.56% | Inflammation: leukocyte recruitment | 13241.85/5.71 | -1.38 | -1.27 |
| IPI00019581 | ★Coagulation factor XII | 3 | 2 | 2.76% | Blood coagulation | 67791.43/8.04 | +1.60 | +1.67 |
| IPI00029168 | Apolipoprotein(a) | 3 | 2 | 0.53% | Lipid transport | 501314.3/5.58 | -1.29 | +1.30 |
| IPI01018212 | Pyruvate kinase isozymes | 2 | 2 | 3.17% | Energy metabolism | 58522.98/7.61 | -1.35 | -1.20 |
| IPI00008318 | ★Ephrin type-A receptor 4 | 1 | 1 | 1.32% | Neurodevelopment | 109858.8/6.21 | -1.50 | -1.38 |
| IPI00641481 | ★Cholesteryl ester transfer protein | 1 | 1 | 2.08% | Cholesterol metabolism | 47786.58/5.64 | +1.36 | +1.28 |
| IPI00878716 | Complement C1s subcomponent heavy chain | 1 | 1 | 16.50% | Immune response/inflammation: complement activation | 11808.05/4.14 | -1.33 | -1.45 |
| IPI00916734 | ★Secreted phosphoprotein 24 | 1 | 1 | 9.16% | Bone morphogenesis | 15143.66/7.71 | -2.59 | -1.73 |
| IPI00922838 | ★Hypoxia up-regulated protein 1 | 1 | 1 | 1.53% | Hypoxia-induced apoptosis regulation | 109704.3/5.87 | +1.40 | +1.26 |
| IPI00924537 | Insulin-like growth factor-binding protein 5 | 1 | 1 | 5.15% | Cell growth regulation | 25480.05/7.04 | +1.49 | +1.34 |
| IPI00945490 | ★Retinoic acid receptor responder protein 2 | 1 | 1 | 6.92% | Retinoid metabolic process | 17812.7/10.25 | -1.47 | -1.24 |

★ Proteins validated by Western blotting.

**Supplementary Table 3. The 25 Differential Proteins Selected for Western Blotting Validation**

| **Accession**  **No. (IPI)** | **Gene Symbol** | **Protein Name** | **Biological Function** | **Antibody Used** |
| --- | --- | --- | --- | --- |
| IPI00004373 | MBL2 | Mannose-binding protein | Immune response/inflammation: complement activation | anti-MBPC (diluted 1:500; Abcam, UK) |
| IPI00007221 | SERPINA5 | Plasma serine protease inhibitor | Blood coagulation | anti-PCI (diluted 1:2000; Abcam, UK) |
| IPI00009028 | CLEC3B | Tetranectin | Blood coagulation: fibrinolysis | anti-TN (diluted 1:2000; Abcam, UK) |
| IPI00019576 | F10 | Coagulation factor X | Blood coagulation | anti-FX (diluted 1:1000; Abcam, UK) |
| IPI00022391 | APCS | Serum amyloid P component | Inflammation: acute-phase response | anti-SAP (diluted 1:1000; Abcam, UK) |
| IPI00032179 | SERPINC1 | Antithrombin-III | Blood coagulation | anti-AT3 (diluted 1:2000; Abcam, UK) |
| IPI00166729 | AZGP1 | Zinc-alpha-2-glycoprotein | Immune response/inflammation | anti-ZAG (diluted 1:200; Abcam, UK), |
| IPI00215894 | KNG1 | Isoform LMW of kininogen-1 | Blood coagulation: platelet degranulation | anti-KNG1 (diluted 1:5000; Abcam, UK) |
| IPI00218732 | PON1 | Serum paraoxonase | Lipid metabolism | anti-PON1 (diluted 1:2000; Abcam, UK) |
| IPI00219583 | SHBG | Sex hormone-binding globulin isoform 3 precursor | Primary spermatocyte growth | anti-SHBG (diluted 1:1000; ABNOVA, Taiwan) |
| IPI00292946 | SERPINA7 | Thyroxine-binding globulin | Thyroid hormone-binding | anti-TBG (diluted 1:500; Abcam, UK), |
| IPI00935408 | CFI CFI protein | CFI | Immune response/inflammation: complement activation | anti-CFI (diluted 1:2000; Lifespan Biosciences, WA, USA) |
| IPI00953689 | AHSG | Alpha-2-HS-glycoprotein | Skeletal system development | anti-AHSG (diluted 1:500; Abcam, UK) |
| IPI00171678 | DBH | Dopamine beta-hydroxylase | Catecholamine biosynthesis | anti-DBH (diluted 1:1000; Abcam, UK) |
| IPI00021855 | APOC1 | Apolipoprotein C-I | Lipid transport | anti-APOC1 (diluted 1:1000; ABNOVA, Taiwan), |
| IPI00022389 | CRP | C-reactive protein | Inflammation: acute-phase | anti-CRP (diluted 1:1000; Abcam, UK) |
| IPI00552578 | SAA1 | Serum amyloid A-1 protein | Inflammation: acute-phase | anti-SAA1 (diluted 1:2000; Abcam, UK) |
| IPI00022731 | APOC4 | Apolipoprotein C-IV | Lipid transport | anti-APOC4 (diluted 1:1000; ABNOVA, Taiwan) |
| IPI00027462 | S100A9 | Protein S100-A9 | Inflammation: leukocyte recruitment | and anti-S100A9 (diluted 1:2000; Abcam, UK) |
| IPI00019581 | F12 | Coagulation factor XII | Blood coagulation | anti-FXII (diluted 1:2000; ABNOVA, Taiwan) |
| IPI00008318 | EPHA3 | Ephrin type-A receptor 4 | Neurodevelopment | anti-EPHA4 (diluted 1:2000; Abcam, UK) |
| IPI00641481 | CETP | Cholesteryl ester transfer protein | Cholesterol metabolism | anti-CETP (diluted 1:2000; Sigma, USA) |
| IPI00916734 | SPP2 | Secreted phosphoprotein 24 | Bone morphogenesis | anti-SPP (diluted 1:1000; Santa Cruz, USA) |
| IPI00945490 | RARRES2 | Retinoic acid receptor responder protein 2 | Retinoid metabolic process | anti-RARRES2 (diluted 1:2000; Sigma, USA) |
| IPI00922838 | HYOU1 | Hypoxia up-regulated protein 1 | Hypoxia-induced apoptosis regulation | anti-HYOU1 (diluted 1:2000; Sigma, USA), |
